# Notes from Dental Practice

**Published:** 1869-07

**Authors:** James B. Hodgkin

**Affiliations:** Alexandria, Va.


### ARTICLE III.

### *Notes from Dental Practice.*

### *Calcified Pulp of a Superior Molar.*

By James B. Hodgkin, D.D.S., Alexandria, Va.

In this case the patient, a lady, called with the left superior second molar aching, and so very sensitive, as to permit but little handling. The tooth had lost its antagonist, and had descended considerably below its fellows in the arch. The crown of the third molar lay against the posterior approximal surface of the affected tooth where the decay was situated so as almost completely to hide the cavity. The patient was very nervous from long suffering. The cavity was diseased as thoroughly as possible, and Welch's nerve paste applied, covered and secured by a pellet of cotton saturated with sandarac varnish, and the patient was requested to call again the next day. On her reappearance the sensitiveness and pain had much abated, but on attempting to excavate the cavity, some considerable uneasiness was manifested, and as the pulp did not appear to be devitalized, a second application of the nerve paste was decided on.

There seemed in the cavity some obstacle in the way of its ready entrance, the nature of which it was difficult to discover from the unfavorable location, as described. The application made as determined on, the patient was dismissed with instructions to call the day following. She failed to make her appearance however, at the appointed hour, and nothing was heard from the case until about ten days after she entered the office stating that she had been hindered from calling sooner on account of sickness. Pain had impelled her to seek relief, and as the tooth was found on examination to be in a bad condition,—acute periostitis with considerable inflammation of the surrounding parts,—it was resolved to yield to her request, and extraction was resorted to. On breaking open the offending organ the cause of the difficulty in the way of a free entrance into the cavity was made man fest. The entire pulp, with the exception of a small investing membrane was calcified. The adherent portion or point from which the growth proceeded, was at the top of the pulp cavity, opposite the bifurcation of the roots. The uncalcified portion of the pulp very nearly completely invested the adventitious growth, and did not much exceed in thickness a sheet of ordinary letter paper. The case is sufficiently unique to excite curiosity.

*A Singular Case.*

A few days ago the writer was called on to extract the roots of a right inferior permanent molar, for a boy of 10 years. The crown of the tooth was entirely gone, there was considerable swelling about the jaw On examination of the extracted roots a large abscess was seen on the anterior root, and from the nerve canal at the apex of the posterior root a straw (a common broom straw) projected nearly one-fourth of an inch, the other end of the straw was broken off level with the upper or crown end of the root. The opening at the apical foramen was large and the roots both much blackened by necrosis. A very considerable haemorrhage followed.

A case a little unusual occurred also in the practice of the writer a few months ago. Patient, a lady with a necrosed lower incisor, the abscess from which discharged from an opening in the point of her chin, which was a long one. On extracting the tooth there was haemorrhage from this fistulous opening.

---

